# miRNAs regulate immune response and signaling during hepatitis C virus infection

**DOI:** 10.1186/s40001-018-0317-x

**Published:** 2018-04-18

**Authors:** Huange Zhu, Yan Geng, Qian He, Miaoxian Li

**Affiliations:** Clinical Laboratory of The Second Affiliated Hospital of Xi’an Jiaotong University (Xibei Hospital), #157 Xiwu Road, Xincheng District, Xi’an, 710004 China

**Keywords:** Hepatitis C virus (HCV), miRNA, Immune response, Signaling pathway

## Abstract

Hepatitis C is one of the most common types of viral hepatitis that impair human health. At present, there is still no effective specific therapy for hepatitis C virus infection. As host immunity is an important mechanism to defend against or clear infections, the interactions between the virus and the host immune response are crucial to the progress of the disease. Of note, hepatitis C virus infection has been reported to regulate cellular miRNAs, which play significant roles in many processes, including infection and immunity. In this review, we describe how miRNAs regulate the host immune response to hepatitis C virus via complex signaling pathways.

## Background

Hepatitis C virus (HCV), an RNA virus in the family *Flaviviridae*, causes one of the most common types of viral hepatitis. Remarkably, the incidence of viral hepatitis continues to increase, despite progress in controlling many other infectious diseases. The primary modes of HCV transmission are unsafe blood transfusion, injection drugs abuse, and maternal-neonatal. Like other viruses, HCV provokes innate and adaptive immune responses that enable the host to resist infection. When such responses fail, the virus replicates and induces acute or chronic hepatitis, as seen both in patients infected with HCV and hepatitis B virus (HBV). Although both HCV and HBV infection induce acute and chronic liver disease, they have profoundly different natural histories and outcomes. Vertical transmission of HBV from mother to neonate always results in chronic hepatitis; instead, HBV infection during adulthood typically results in lifelong protective immunity [1]. By contrast, HCV infection readily establishes chronic hepatitis in 60–80% of infected adults [1]. Today, there are about 360 million and 180 million cases of chronic hepatitis B and C infection, respectively [2]. Due in part to impaired immunity, HCV infection is more likely to progress to chronic hepatitis C, a leading cause of liver cirrhosis and, subsequently, hepatocellular carcinoma. In chronic hepatitis C, HCV can co-exist with a permanently host immune system for decades. Indeed, about 80% of hepatocellular carcinoma cases are related to chronic virus infection [2]. In turn, hepatocellular carcinoma is the most common form of liver cancer, and accounts for the sixth and third largest cases of cancer morbidity and lethality in the world, highlighting the significance of HCV as a worldwide public health issue.

The traditional therapy for HCV infection is a combination of interferon (IFN) and ribavirin. However, this regimen has low cure rate, and is effective only for a fraction of patients [3]. On the other hand, direct-acting anti-viral, which is still considered as a new therapeutic approach, has a higher cure rate [4], but high cost and drug resistance have limited wide use. Hence, there is still no effective specific therapy that can be widely used against HCV. Since the host immunity is vital in the process of HCV infection, analysis of the host immune response is crucial to identify new targets for therapy, as well as to understand the specific mechanisms driving HCV infection.

Of note, expression or dysregulation of microRNAs (miRNAs) are associated with an array of disease states, including diabetes, neurological, blood, and immune disorders [5, 6]. miRNAs are small noncoding RNAs, which, along with long noncoding RNAs, comprise a class of RNAs that do not encode proteins. Mature miRNAs are about 18–25 nucleotides, and are generated via RNA polymerase II/III-dependent transcription and subsequent processing by RNase III [7]. In 1993, Ambros and collaborators demonstrated that the miRNA lin4 inhibits LIN14 protein expression, but not mRNA expression [8]. Since then, miRNAs have become the focus of extensive mechanistic and functional studies.

miRNAs play important roles in growth and development. For instance, some miRNAs are specifically expressed in embryonic stem cells, and probably help maintain pluripotency [9]. miRNAs also mediate late embryo gastrulation, heart development, neurogenesis, and other events [10]. In addition, miRNAs modulate metabolism by regulating insulin release, cholesterol metabolism, and other processes. Finally, miRNAs regulate the expression of target mRNAs at transcription or post-transcription, such as by inhibiting translation or by altering mRNA stability.

## miRNAs regulate the immune response to HCV

Organisms are constantly exposed to pathogens in the environment, which are subsequently cleared by the host’s innate and acquired immunity. Although innate immunity is the first line of defence against pathogens, acquired immune responses are also necessary for full protection. As such, HCV has evolved escape strategies to establish persistent infections. Chronic hepatitis, cirrhosis, hepatocellular carcinoma, and other diseases associated with HCV are closely linked to the host immune response [11]. On the other hand, a growing body of evidence indicates that miRNAs shape infection and immunity [6, 12], not only by regulating the differentiation and development of immune cells, but also by mediating the immune response to infection or other disorders. For example, miRNAs suppressed by HCV regulate immune reactions, antigen presentation, cell cycle progression, and lipid metabolism, while miRNAs suppressed by hepatitis B virus regulate cell death, DNA damage and repair, and transcription [13].

The miRNA miR-122 is specifically expressed in the liver, and accounts for approximately 70% of total liver miRNA [14]. miR-122 binds to the 5′-UTR of and stabilizes the HCV RNA genome, and thereby stimulates virus replication [14, 15]. On the other hand, pre-miR-122 or unprocessed single-stranded miR-122 cannot bind to the HCV 5′-UTR, and cannot modulate HCV RNA accumulation. It is essential for miR-122 modulation of HCV translation and RNA accumulation, that pre-miR-122 must be processed by Dicer and TRBP and strand selection of mature duplex miR-122 [16]. Human Dicer and TRBP proteins involve in the biogenesis pathway of miR-122 mature, but they are not needed for the mechanism of HCV RNA accumulation and HCV translation provided by mature duplex miR-122 [16]. Inhibition of miR-122 also boosts HO-1 mRNA expression and reduces HCV RNA expression, highlighting its potential as a therapeutic target [17]. An inverse correlation between miR-122 and biochemical evidences of hepatocyte damage, including alanine aminotransferase, aspartate aminotransferase, and fibrosis, but not viral load, has also been noted in patients with chronic hepatitis C [18], with miR-122 expression consistently downregulated at advanced stages of fibrosis. Decreased levels of miR-122 were also predictive of poor response to therapy with IFN [19]. Strikingly, miR-122 was also shown to impede HCV entry into hepatocytes by binding the 3′-UTR of OCLN mRNA. Accordingly, miR-122 mimics may be more beneficial than miR-122 inhibitors in early infection [20], as low miR-122 levels at the beginning of infection may facilitate HCV entry into hepatocytes. Both miR-122 inhibitors and mimics have advantages in HCV infection, which still needs to have further careful studies to evaluate.

Various relationships between miR-122 and hepatocellular carcinoma have also been noted. Some studies showed that miR-122 is strongly expressed in malignant liver nodules, and may promote tumorigenesis by inhibiting tumour suppressors in hepatocellular carcinoma related to HCV infection [21]. Corollary, other studies indicated that miR-122 is downregulated in hepatocellular carcinoma unrelated to HCV [22]. Collectively, these studies imply that miR-122 plays essential and complex roles not only in HCV infection, but also in hepatocellular carcinoma regardless of HCV status [21].

miR-155, an miRNA linked to inflammatory monocyte activation and a common target of many inflammatory mediators, not only promotes autoimmune inflammation by enhancing the development of inflammatory T-cells [23], but also plays important roles in immune response and HCV infection [24]. For instance, HCV induces miR-155 expression in vitro and in vivo. Accordingly, miR-155 is significantly more abundant in the liver tissue and serum in HCV-infected patients than in uninfected controls [25, 26]. In addition, miR-155 markedly diminishes in patients who successfully clear the virus after treatment, reinforcing the notion that upregulated miR-155 facilitates HCV infection [25]. Indeed, miR-155 regulates the innate immune response by regulating IFNr production from natural killer cells during chronic hepatitis C infection [27], and controls the immune response to HCV in the liver [24].

Similar to miR-155, miR-146a is abundant in peripheral blood mononuclear cells and regulates the inflammatory and immune responses [28]. It has recently been reported that miR-146 was upregulated in HCV-infected cells, human primary hepatocytes and liver tissue of chronic hepatitis C infection patients [28]. On the other hand, decreased miR-146a expression has also been observed in peripheral blood mononuclear cells from patients with chronic hepatitis C. This can partially be explained as the studies analysed patients infected with different genotype HCV. Moreover, miR-146a expression is significantly correlated to serum cholesterol, and well as cholesterol levels in peripheral blood mononuclear cells. This is not unsurprising as the cholesterol metabolism plays a critical role in the HCV life cycle. However, the detail mechanisms in miR-146a expression and cholesterol metabolism, and its inflammatory and immune roles remain to be elucidated.

The role of miR-130a in HCV infection is similarly complex. miR-130a expression in the HCV-infected liver is much higher than in control, and miR-130a knockdown also inhibits HCV RNA replication in hepatocytes by inducing IFITM1 expression [29], which, in turn, stimulates secretion of IFN. Thus, HCV may persist by up-regulating miR-130a to inhibit IFITM1 and the subsequent innate immune response. On the other hand, miR-130a overexpression was also reported to suppress HCV RNA replication in both the Con1b replicon and in the JFH1-based cell culture system [30]. Further, miR-130a was found to downregulate miR-122 but upregulate proteins that coordinate the host innate immune response, including type I IFN (IFNα/IFNβ), ISG15, USP18, and MxA. Collectively, the data indicate that miR-130a may play dual roles in HCV replication by shaping the host innate immune response.

miR-21 is also upregulated both in liver biopsies and Huh7.5 cells after HCV infection (18). In turn, miR-21 represses the expression of type I IFN and the subsequent anti-viral response to enable the virus to evade the host immune system and replicate [31]. miR-21 also stimulates the proliferation of hepatocellular carcinoma cells [32], perhaps driving disease progression in this manner. Indeed, miR-21 is positively correlated with clinical indices in patients with chronic hepatitis C, including viral load, fibrosis, serum alanine aminotransferase, and aspartate aminotransferase [18]. Moreover, miR-21 directly targets SMAD7, a member of SMADs which was described in details follow-up, in Huh7 cells [18], although the pathologic significance of this relationship is unclear.

Dual specific phosphatases (DUSP) are signature markers of T cell aging, of which DUSP 5 and 6 have been implicated in loss of T cell antigen receptor sensitivity [33]. Remarkably, a drop in miR-181a expression in CD4^+^ T cells derived from HCV-infected patients boosts DUSP 6 expression and CD4^+^ T cell dysfunction [34], suggesting a possible mechanism by which HCV interacts with the cellular immune response to establish infection.

Lipid overload due to HCV infection also boosts expression of miR-27 in the liver, which, in turn, inhibits virus infection and promotes lipid storage. However, this suppressive effect also enables the virus to escape immune surveillance by lowering the viral load [35].

By directly binding the 3′-UTR of Bach1 mRNA, miR-196 suppresses Bach1 expression, stimulates HMOX1 expression, and inhibits HCV gene expression [36]. Surveys showed that circulating miR-196a is significantly lower in patients with chronic hepatitis C, regardless of HCV viral load or alanine aminotransferase levels. This result was attributed to the reduced release of miR-196a from HCV-infected hepatocytes, highlighting miR-196a as a potential biomarker for early diagnosis of chronic hepatitis C [37]. Of note, miR-196a expression can be stimulated in hepatocellular carcinoma cells by exposure to IFNβ [38]. Finally, we note that many other miRNAs and miRNA clusters have been reported to impact HCV infection.

## Signaling pathways regulated by miRNAs during HCV infection

Signaling pathways, which form a highly complex cross-regulated network in cells, are the mechanism by which extracellular molecules transmit signals into cells, trigger a series of enzymatic and biochemical reactions, and ultimately elicit cellular responses. These pathways are tightly regulated as aberrant signaling can have pathophysiological consequences. Accordingly, the various interactions between miRNAs and HCV are closely linked to these pathways.

### Toll-like receptor (TLR) signaling

TLR signaling activates the innate immunity and promotes the development of antigen-specific acquired immunity. There is more than one receptor involved in TLR signaling. TLRs are a family of receptor members, a type of pattern recognition receptors (PRRs). These receptors are widely expressed on the membranes of immune and non immune cells, including dendritic cells, macrophages, natural killer cells and fibroblasts, which is crucial for recognizing or defensing invading pathogens and endogenous ligands. Ligand binding triggers the recruitment and modification of downstream signaling molecules—including MyD88, IRAKs, TAK1, TAB1, TAB2, and TRAF6—to ultimately induce NF-κB nuclear translocation and regulate target gene expression.

HCV infection upregulates miR-21 expression and miR-21 suppresses the production of type I IFN [31]. Moreover, miR-21 overexpression and inhibition in Huh7 hepatocytes can decrease and increase the expression of several TLR pathway effectors, including MyD88, IRAK1, IRAK4, and TRAF6—of which MyD88 and IRAK1 are direct targets of miR-21 and can modulate type I IFN production. Thus, HCV infection upregulates miR-21 and represses IFNα signaling through MyD88 and IRAK1 [31], both of which link TLR and IL-1R to downstream signaling molecules. In turn, repression of type I IFN helps the virus evade the host immune system, suggesting that TLR signaling is one mechanism by which miRNAs can regulate virus invasion. While these founds of miR-21 are studied in hepatocytes which are less TLR expressers than Kupffer cells, the complex roles and mechanisms still need further studies. In addition, there is an inverse correlation between miR-125b levels and cytokine expression after HCV core protein challenge [39]. Studies have shown that miR-125b expression is downregulated and cytokine production was upregulated in THP-1 cells, a human monocytic leukemia cell line, following HCV core protein stimulation. Furthermore, forced miR-125b expression can abolish the cytokine production induced by HCV core protein by inhibiting NF-κB p65, ERK, and p38 phosphorylation. Collectively, this evidence suggests that miR-125b may negatively regulate HCV-induced immune responses by targeting TLR2/MyD88 signaling in monocytes [39]. And it still should be noticed that forced HCV core protein expression may not recapitulate the exact process and mechanism during HCV-infection. These works give us new sight and information that may be crucial for HCV infection, which still need further studies to verify.

### JAK/STAT signaling

The Janus kinase/signal transducer and activator of transcriptions (JAK/STAT) pathway is frequently engaged by cytokines and growth factors that regulate cell proliferation, differentiation, apoptosis, inflammation, and other processes. Indeed, IFN activates this pathway to elicit an anti-viral response and JAK/STAT pathway activation may lead to higher IFN therapy responses. On the other hand, JAK/STAT signaling is suppressed by suppressors of cytokine signaling (SOCS) proteins, of which SOCS3 is one of the most potent. Therefore, both JAK/STAT signaling activity and SOCS proteins may play functional roles in IFN resistance in HCV-infected patients.

Notably, silencing miR-122, which is strongly expressed upon HCV infection and is predictive of the response to IFN therapy [14, 19], also suppresses SOCS3 expression by methylation of its promoter, and thereby boosts STAT3 activation [40] to enhance the response to IFN therapy. miR-19a, a member of the miR-17-92 cluster that is dysregulated in HCV infection [41], probably has similar clinical value, as it suppresses SOCS3 expression to enhance IFNα and interleukin-6 signaling via STAT3, and thus activates JAK/STAT signaling [42]. HCV infection also stimulates miR-373 expression, and thereby impairs JAK/STAT signaling by directly targeting JAK1 and IFN-regulating factor 9 [43]. Taken together, these observations imply that JAK/STAT signaling and SOCS proteins may underlie IFN resistance in HCV patients.

### TGF-β1/SMADs signaling

Transforming-growth factor-β (TGF-β) is synthesized by many cells and has a fibrogenic capacity by serving as a chemotactic for fibroblasts, stimulating fibroblast proliferation, and increasing extracellular matrix protein synthesis. In addition, TGF-β is critical for liver fibrogenesis induced by virus infection or other factors, and also has anti-inflammatory and immunosuppressive effects upon binding its cognate receptors.

SMADs are a family of intracellular regulatory proteins that regulate signaling downstream of TGF-β receptors. SMADs can be divided into three classes, including receptor-regulated SMADs, common-mediator SMAD and inhibitory SMADs. SMAD7 is one inhibitory SMAD, which can block the activation of receptor-regulated SMADs and common-mediator SMADs. Notably, upregulation of miR-21 in HCV and hepatocellular carcinoma patients represses a luciferase reporter containing the 3′-UTR of SMAD7 and increases luciferase production from a TGF-β reporter [18]. On the other hand, SMAD7 is a feedback inhibitor of TGF-β and its expression is induced by TGF-β. Collectively, these observations highlight a potential relationship between miR-21 and TGF-β1, possibly via SMAD7, that could drive fibrogenesis in chronic hepatitis C. Moreover, miR-192 increased by HCV infection can directly upregulate TGF-β1 expression, suggesting that it is also as a major factor mediating HCV infection-associated fibrogenesis [44].

### Wnt signaling

Wnt is a highly conserved pathway and a key regulator of cell proliferation. Ad-HCV core adenovirus infection can downregulate miR-152 and upregulate the Wnt/β-catenin effector Wnt1 in HepG2 cells. Notably, HCV downregulates miR-152 [45], which directly targets the *WNT1* 3′-UTR, thereby relieving the suppression and inducing Wnt1-mediated signaling that promotes in proliferation, G1-S transition, and colony formation in HepG2 cells. Nuclear localization of β-catenin—another marker of Wnt/β-catenin activity—is also much higher in HCV-infected and hepatocellular carcinoma patients than in healthy controls. For instance, miR-155 over-expression increases β-catenin nuclear localization in Huh7 cells [25], suggesting that it may regulate the immune response to HCV and thereby link HCV to cancer through Wnt/β-catenin signaling.

### PI3K/Akt signaling

Phosphatidylinositol 3-kinase and Akt/Protein Kinase B (PI3K/Akt) signaling is a classic and frequently engaged intracellular signal transduction pathway that mediates physiological and pathological processes—including metabolism, cell proliferation, cell survival, and angiogenesis in response to extracellular signals—via serine and/or threonine phosphorylation. miR-491, identified to be downregulated by HCV infection in Huh7 cell line, can enhance HCV replication both in HCV replicon cells and in cell culture-infectious HCV-infected cells. The study finds that the effect of miR-491 on HCV replication can be abolished by inhibiting PI3K, which indicates that augmentation of HCV replication by miR-491 is dependent on the PI3K/Akt pathway [46]. On the other hand, HCV also engages the PI3K pathway to activate miR-27, resulting in hepatic steatosis [47].

### Other signaling pathways

Many other miRNAs have been predicted to regulate the NF-κB, MAPK, or other signaling during HCV infection. NF-κB is an important signaling pathway downstream of TNF-α, which promotes inflammation, infection, immune processes, and cell apoptosis. It has been suggested that the non-structural protein 5A (NS5A), encoded by HCV RNA genome, can decrease miR-503, which is abnormally expressed in various cancers and may play complicated and tissue-specific roles in cancer, and increase bcl-2 by inhibiting NF-κB activation [48]. This can be a potential mechanisms contributing to HCV infection, as NS5A plays a critical role in HCV replication. Moreover, miRNA-449a in liver biopsies is downregulated in chronic HCV infected patients, but not in patients with alcoholic or non-alcoholic liver disease, and the downregulation of miRNA-449a promotes TNFa-mediated activation of YKL40 through NOTCH signaling pathway [49]. On the other hand, miR-155 regulates IFNγ production in natural killer cells via Tim-3 to balance immune clearance and immune injury during chronic hepatitis C [27].

## Conclusions

HCV readily establishes chronic infection in adults. Virus genome factors, host innate immune response, and virus escape from the adaptive immune response are crucial to this process. Accordingly, clear understanding of the host immune response to HCV will illuminate specific mechanisms of pathogenesis, and promote the development of vaccines and immunotherapies. The body of evidence now suggests that miRNAs are key elements in HCV infection and the subsequent immune response, as well as in other physiological and pathological processes. Indeed, many miRNAs are dysregulated during HCV infection, and directly alter the host immune response or the replication of the virus by engaging various signaling pathways (Table 1). Contradictory miRNA activities have been described, highlighting complex roles in HCV infection and host immunity. Hence, specific roles and mechanisms require further verification, although these can now be predicted by bioinformatics. All the studies expressed demonstrate that miRNA expression is highly regulated in response to HCV infection and plays a significant role in virus infection and disease pathophysiology; however, the more detailed mechanisms in miRNA regulation and HCV infection, and disease pathophysiology still require further investigation.

**Table 1:** miRNAs and the related immune responses and signaling pathways during hepatitis C virus infection

miRNAs	Related pathways	Immune responses and roles	References
miR-122	JAK/STAT signaling	Stabilize the HCV RNA genome and stimulate virus replication Predict response to therapy with IFN Impede HCV entry into hepatocytes	[14–22]
miR-155	Wnt signaling Tim-3 signaling	Enhance the development of inflammatory T-cells Promote autoimmune inflammation Regulate IFNr production from NK cells Increase β-catenin nuclear localization in Huh7 cells	[23–27]
miR-146	TLR signaling	Regulate the inflammatory and immune responses Correlate with cholesterol metabolism	[28]
miR-130a	JAK/STAT signaling	Inhibit IFITM1 and the subsequent innate immune response Play dual roles in HCV replication by shaping the host innate immune response	[29, 30]
miR-21	TLR signaling TGF-β1/SMADs signaling	Repress the expression of type I IFN and the subsequent anti-viral response Target SMAD7 Stimulate the proliferation of hepatocellular carcinoma cells	[18, 31, 32]
miR-181a	MAPK/ERK signaling	Decrease DUSP 6 expression and CD4^+^ T cell dysfunction	[34]
miR-27	PI3K/Akt signaling	Inhibit virus infection and promotes lipid storage Enable the virus to escape immune surveillance	[35, 47]
miR-196	JAK/STAT signaling	Suppress Bach1 expression, stimulates HMOX1 expression, and inhibits HCV gene expression.	[36–38]
miR-125b	TLR signaling	Abolish the cytokine production	[39]
miR-19a	JAK/STAT signaling	Enhance IFNα and interleukin-6	[42]
miR-192	TGF-β1/SMADs signaling	Upregulate TGF-β1 expression Mediate HCV infection-associated fibrogenesis	[44]
miR-152	Wnt signaling	Target the *WNT1* 3′-UTR Regulate proliferation, G1-S transition, and colony formation in HepG2 cells	[45]
miR-491	PI3K/Akt signaling	Enhance HCV replication	[46]
miR-449a	NOTCH signaling	Inhibit TNFa-mediated activation of YKL40	[49]

## Abbreviations

HCV: hepatitis C virus
IFN: interferon
miRNA: microRNAs
DUSP: dual specific phosphatases
TLR: toll-like receptor
JAK/STAT: Janus kinase/signal transducer and activator of transcriptions
SOCS: suppressors of cytokine signaling
TGF-β: transforming-growth factor-β
PI3K/Akt: phosphatidylinositol 3-kinase and Akt/protein kinase B
NS5A: non-structural protein 5A
